# Annotations

**Published:** 1908-06-06

**Authors:** 


### Annotations.

### L'Entente Cordiale Médicale.

The visit of the President of the French Republic to the French Hospital on May 28 was a subject of much gratification to the authorities of that institution. In spite of the shortness of his stay in London and the large number of his engagements, the President managed to fulfil his promise to the hospital and show his practical interest in this international charity, wherein medical men of both races work side by side for the benefit of those of the sick poor of London who speak the French language. The French Hospital is a permanent symbol of the friendly understanding and co-operation between the two nations which is now known on both sides of the Channel as *l'entente cordiale*; and M. Fallières' visit has not only increased his great personal popularity with Englishmen, but has also lent material aid to the work of a deserving institution. In addition to his present of £200 to the poor of London, the President before leaving made handsome gifts to the French Hospital and to several other French charities situated in this country. It is interesting to note, in connection with *l'entente cordiale médicale*, that Sir Dyce Duckworth's recent lecture before French medical men in Paris is to be followed by the delivery at the Royal College of Physicians of London of a lecture by Dr. Fulgence Raymond, of the Salpêtrière Hospital, Paris. As we announced last week, Professor Raymond is to receive the honorary degree of Doctor of Science from the University of Oxford on June 24. The subject of his lecture will be "Les Maladies dites Familiales," and it will be delivered at the College of Physicians on Monday, June 22, at five o'clock.

### The Prevention of Crime.

The Home Secretary recently drew the attention of the House of Commons to the fact that civilised countries are nowadays paying special attention to the prevention rather than to the punishment of crime. The occasion of this remark was the introduction of a Bill to make better provision for the prevention of crime, by the reformation of young offenders and the prolonged detention of habitual criminals. It really seems, therefore, that the Government of this country is awaking to the necessity for the scientific, as opposed to the vindictive or the sentimental treatment of the criminal. The first part of Mr. Gladstone's Bill proposes the establishment of State reformatories for young offenders, conducted on the well-known Borstal principle. This scheme will not come as a great surprise, since it has already been foreshadowed to some extent. The second part of the Bill is, in our opinion, a real advance in legislative efforts. It may meet with conscientious opposition at first; but we believe that public opinion is gradually coming round to the necessity for scientific methods in public affairs, and that this measure will not only become law, but that it will do good. The essence of it is the segregation of the habitual able-bodied criminal, who takes to crime by preference, refuses work, and laughs at the present system of imprisonment. This habitual offender is to be sentenced to an additional term of detention after the completion of his ordinary sentence of penal servitude. The Bill provides for the building of a special place for such detention in the Isle of Wight, wherein the ordinary prison rules would be modified, the discipline would be less rigorous, and good conduct would meet with an adequate and progressive reward. No one would be passed on to this place unless he had previously been convicted of at least three serious crimes, and he would have an unqualified right of appeal from the sentence of the Court. Moreover, it would be open to him to secure his own discharge on reasonable conditions. He would not be treated as a hopeless offender, but from the first every effort would be made to reclaim him. As the Home Secretary wisely said, hope and not fear is the best antidoe for criminality. We believe that the passing of this measure would be an important step towards the solution of a difficult problem.

### Midwives and Cancer.

The official attention of the Central Midwives Board has recently been drawn by the British Medical Association to the question of the recognition of uterine cancer by midwives, and it is good news to hear that the Board was able to reply that the subject is already under consideration, and that a leaflet, prepared by the Chairman (Dr. Champneys) is about to be sent to midwives, teachers, and local supervising authorities. It is not to be expected or desired that unregistered persons of quasi-medical attainments should be instructed in the physical signs of any tumour, innocent or benign. But it is very much to be hoped that a spread of information as to the early symptoms of carcinoma of the uterus and cervix may prove a valuable means of bringing surgical treatment to bear on the sufferers from these conditions at an earlier and therefore a far more hopeful stage than is at present the case. A midwife who confines her energies entirely to parturient women is not likely to come across cases of carcinoma except at the longest intervals, but a lying-in chamber is one in which the patients' friends often gossip freely on their troubles, pelvic and otherwise, with the attendant midwife. If midwives can be trained to understand the vital necessity for referring to a doctor all those who mention in their hearing a certain train of symptoms, in all probability a good many cases that have now to be rejected by surgeons as inoperable and abandoned to their fate will be saved to the community and restored sound to their friends. A good deal has been heard of quite recent years about the shortcomings of the medical profession in respect of the early diagnosis of uterine cancer. We are quite prepared to admit that there has been a fair amount of justice in some of the strictures that have been passed, but the profession as a whole is now much more alive to the importance of the point, and the principal obstacle to progress is now certainly the ignorance and carelessness and reluctance of the laity rather than the remissness of their medical attendants.

